# Assessment of guideline adherence in breast cancer management among oncologists in Nigeria

**DOI:** 10.3332/ecancer.2021.1294

**Published:** 2021-09-23

**Authors:** Bolanle C Adegboyega, Adewumi O Alabi, Adedayo O Joseph, Nwamaka Lasebikan, Luther A Agaga, Kehinde O Ololade, Anthonia C Sowunmi

**Affiliations:** 1 Department of Radiotherapy, Lagos University Teaching Hospital, Idi Araba Mushin, Lagos, 234, Nigeria; 2 Department of Radiation Biology, Radiotherapy and Radiodiagnosis, College of Medicine, University of Lagos, Idi Araba Mushin, Lagos, 234, Nigeria; 3 NSIA-LUTH Cancer Centre, Lagos University Teaching Hospital, Idi Araba Mushin, Lagos, 234, Nigeria; 4 Department of Radiation Oncology, University of Nigeria Teaching Hospital, Ituku-Ozalla, Enugu, 234, Nigeria; 5 Lagos State University Teaching Hospital, Oba Akinjobi Way, Ikeja, Lagos, 234, Nigeria

**Keywords:** breast cancer, treatment guidelines, oncologist, adherence, Nigeria

## Abstract

**Background:**

Breast cancer management is evolving by the day and new discoveries is shifting the scale to more positive result mostly in developed countries and this is being reported and updated in the treatment guidelines to bridge the knowledge gaps and allow for global standardised management protocol. This study assessed the adherence to the breast cancer guideline use among oncologists in Nigeria, reviewing the commonly used guidelines, factors for the choice, effects on treatment and barriers to usage.

**Methodology:**

A proforma was sent by mail to the oncologist in Nigeria assessing their socio-demographic characteristics, knowledge of guidelines, use of guidelines, barriers to use of guidelines and benefits of guideline use and all the those that completed the survey within 1-month period were included in the study.

**Results:**

A total of 109 oncologist responded to the survey with mean age of 42 years, mean year of oncology practice was 10 years. Sixty-four percent were consultants and 38% residents-in-training. All respondents were aware of breast cancer guidelines and 92.2% had used it in treatment decision making. The commonest used being National Comprehensive Cancer Network guideline in 87.4% and 82.6% had a choice guideline/institution adopted. The major reason for referring to a choice guideline by 66% of respondents was to gain access to evidence-based results and the major barrier to guideline use in 56% of cases was non compatibility with available resources.

**Conclusion:**

The study revealed high level of adherence to breast cancer guideline use among oncologists in Nigeria but there is need for more awareness about the locally developed ones like sub-Saharan adapted version and institutional based breast cancer treatment guidelines so as to address the barrier of disparities in target population and resources availability.

## Introduction

Breast cancer is the most common cancer among women worldwide and the second most common cancer in both sexes [1]. Previously thought to be a disease of the developed world; however, 56% of breast cancer cases and 63% of deaths from breast cancer occurred in less-developed countries by 2011 [2]. In Nigeria, breast cancer is the commonest cancer overall as well as being the commonest cancer in females with an incidence of 22.7% and mortality of 16.4% among all cancers in 2018 [3].

The Institute of Medicine defines clinical practice guidelines as statements that include recommendations intended to optimise patient care that is informed by a systematic review of evidence and an assessment of the benefits and harms of alternative care options [4]. These guidelines were established to improve high-quality evidence-based care of patients and to prevent undesirable practice variations [5]. The Guidelines International Network currently contains more than 6,400 guidelines from 85 countries created by reputable guideline development organisations that are based in developed countries [6]. These treatment guidelines are used globally by health care providers across all fields of medicine to ensure quality care, standardised care and cost control; however, its usage varies based on regions, available facilities, patient demographics, health care funding, disease presentation, health care personnel and expertise in providing health care [7]. This study was intended to access the standard of breast cancer care being provided by oncologist in Nigeria based on the level of adherence to these guidelines and barriers to their use as well as reasons for any variations that might be identified.

## Methodology

This study is a non-interventional, cross-sectional review. It was approved by Lagos University Teaching Hospital (LUTH) Health Research and Ethics Committee and the study sample was drawn from all oncology specialists and trainees in Nigeria. It was an online survey conducted between March and April 2020, a google form was designed comprising of a consent form and questionnaire and sent by mail to all the identified oncologists in Nigeria including reminders to non-respondents, to maximise the response rate. The questionnaire was pre-tested among few oncologists to address issues and biases raised before the final survey. Consent form was a pre-requisite to completing the survey and confidentiality of the response was ensured.

The form is divided into five sections (A–E) to obtain data on:

- Socio-demographic characteristics of the respondents; this includes age, sex, marital status, cadre, speciality, number of years of practice, state of practice, location of practice, foreign training and duration of training.
- Knowledge of guidelines: participants were asked specific questions on known breast cancer guidelines, and how they got to know about it.
- Use of guidelines: choice guideline, institution adopted guideline and the reason(s) for referring to guidelines.
- Barriers to use of guidelines.
- Benefits of guideline use.

The result was analysed using Statistical Package for Social Sciences version 21. Univariate analyses were presented in tables and diagrams while association analyses were done using Chi square and analysis of variance with a significant *p*-value of ≤0.05.

## Results

### Oncologist demographics

A total of 103 respondents completed the online survey and they were mostly (41.7%) between ages 40 and 49 years (mean age was 41.5 years), male (68.9%) and married (92.2%). Sixty-two percent were consultants and the rest were residents. Of the respondents, radiation and clinical oncologists accounted for 75.7% while the rest were surgical oncologists. The respondents reported a median of 10 years of oncology practice (ranging between 5 and 10 years in 34.0% of this group) and 69.9% of them practice in University Hospitals. Just over half (52.4%) of the respondents had had trainings abroad and trainings mostly lasted between 1 and 5 months. The commonest states of practice were Lagos (35.9%), Federal Capital Territory (FCT) Abuja (12.6%) and Oyo state (9.7%) (Table 1a and b).

### Basic awareness and usage

**Figure 1.**
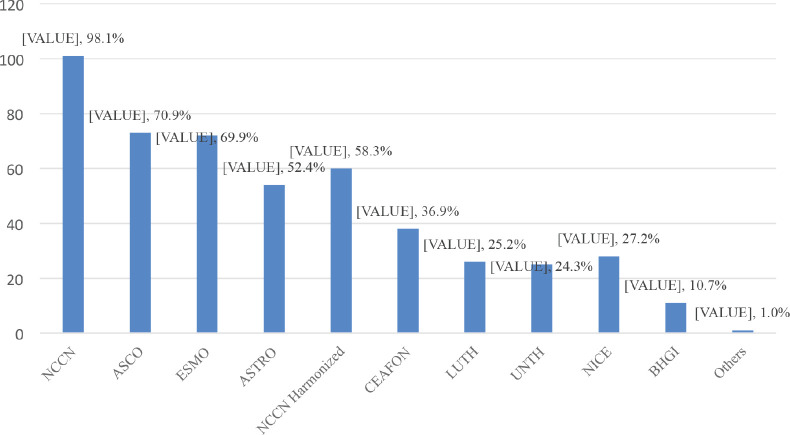
Breast cancer guidelines known by respondents.

**Figure 2.**
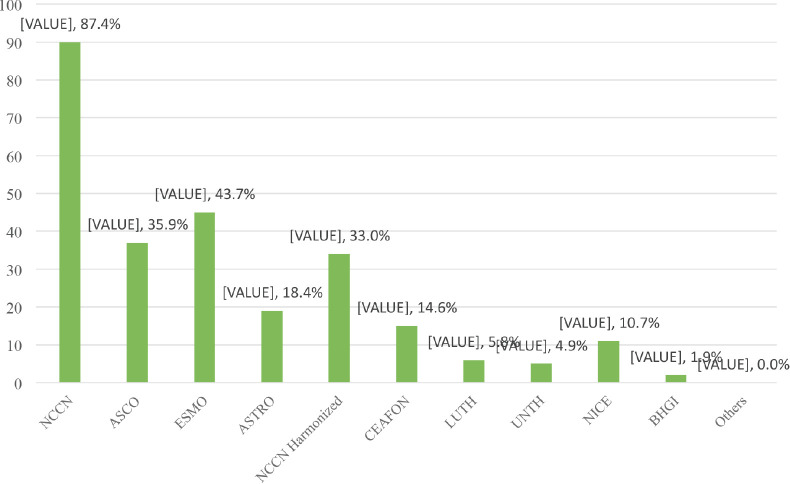
Breast cancer guidelines used by respondents.

**Figure 3.**
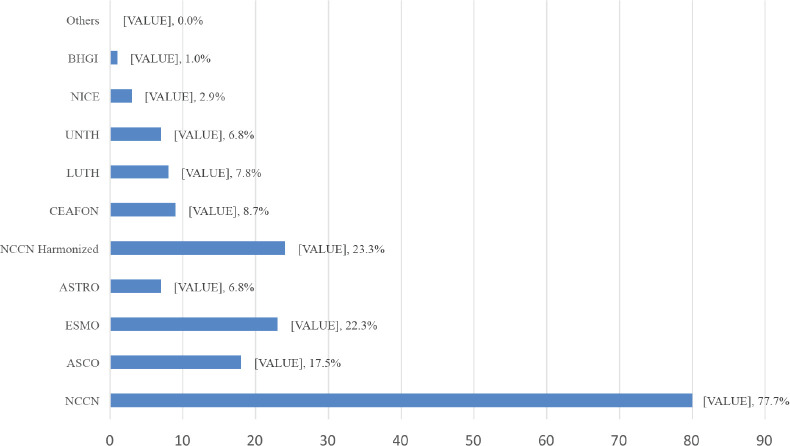
Breast cancer guidelines routinely used/adopted in respondents’ institutions.

All (100%) the respondents were aware of breast cancer management guidelines. These guidelines included; National Comprehensive Cancer Network (NCCN) guidelines (98.1%), American Society of Clinical Oncology (ASCO) guidelines (70.9%) and European Society of Medical Oncology (ESMO) guidelines (69.9%) (Figure 1). Some of the respondents first learnt about these breast cancer guidelines from their consultants/mentors (37.9%) (Table 2). Majority of the respondents had used breast cancer guidelines to make treatment decisions (92.2%). The commonest being; NCCN guidelines (87.4%), ESMO guidelines (43.7%) and ASCO guidelines (35.9%) (Figure 2). Majority (81.6%) of the respondents indicated that they had one or more breast cancer guidelines routinely used and/or adopted in their institution which includes; NCCN guidelines (77.7%), NCCN Harmonised Guidelines for Sub-Saharan Africa (23.3%) and ESMO (22.3%) (Figure 3).

### Practice pattern

Respondents were asked specific questions to determine the reasons for their choices, benefits and barriers to guideline use. The frequency of use among respondents were; always (27.2%), often (38.8%), sometimes (24.3%) and rarely (9.7%) (Table 3).

The main reasons for referring to a guideline was to access evidence-based results (66.0%) while other reasons were identified during multidisciplinary team (MDT) review (41.7%) (Table 3). Respondents used these guidelines more in government hospitals (81.6%) than private hospitals (18.4%). Its use in private setting was mostly because patients there could afford the recommendations (94.7%) and practitioners tried to provide top notch services (47.4%) (Table 4).

Identified perceived benefits of using breast cancer guidelines among the participants included; standardised care (92.2%), evidence-based management (89.3%) and improved patient outcome (70.9%) (Table 5). This is further enhanced by adoption of institution-based guidelines/protocol (73.8%), affordable cancer care/health insurance coverage (69.9%) and improved accessibility to guidelines (68.0%) (Table 5).

The barriers to the use of breast cancer guidelines included; non-compatibility with available resources (56.3%), hindering individualised care in some unique circumstances (26.2%) and its complexity (24.3%) (Table 6).

Statistically significant associations between socio-demographic characteristics and awareness and use of breast cancer guidelines are shown in Table 7. Guideline usage was more frequent among the middle-aged doctors (30–49 years) (Table 7a), the married (Table 7b), consultants (Table 7c), Radiation oncologists (Table 7d) and those that have had additional trainings abroad compared to their counterparts who did not have training abroad (Table 7e).

## Discussion

This study characterised the oncologists in Nigeria. The greater percentage were in the South-Western and North-Central compared with other parts of the country and there were many more Clinical and radiation oncologists (75.7%) than Surgical Oncologists (24.3%). There are three levels of health system in Nigeria, namely Primary health centres (PHC), which is supposed to take care of the community, Secondary/General hospital meant to take referrals from the PHC and Tertiary/Teaching hospital where referrals of complicated cases are seen. MDT meetings are also held in some tertiary hospitals in Nigeria. Nearly all the oncologists work in government/public hospitals (69.9%), mainly at the tertiary centres. There is minimal access to information technology in the hospitals save for few individuals with personal laptops and mobile phones with which they are able to access the guidelines.

This study however confirmed that 40 (39%) of the respondents often make use of the breast cancer guideline use. These cut across the various cadre and specialities but with statistically significant use among consultants, who are considered the opinion leaders in the team, thus suggesting that adherence to guideline use increases with the number of years of practice. A study by Tunis *et al* [9] reported that familiarity with the guidelines improves use and eventual building of confidence in the treatment guideline. The study also found more usage among the Radiation and Clinical oncologists than the Surgical oncologists, which is comparable with an earlier study by Jagsi *et al* [10] which revealed that more medical oncologist than surgical oncologists are more aware of NCCN guidelines, agreed with the recommendation and use it for most of their patients. Other significant differences reported with more adherence included being middle aged and having had overseas trainings. This study did not find any statistically significant difference in adherence to the guideline in gender and geographical location of practice contrary to an earlier report by Jagsi *et al* [10] implying that no knowledge gap exists among oncologists in Nigeria irrespective of regional location.

The study revealed that the most common breast cancer guideline used among oncologists in Nigeria is the NCCN guideline (87.4%) followed by the ESMO (43.7%) and ASCO (35.9%) guidelines in that order. Some oncologists refer to more than one guideline in decision making. Dillmon and Kerr *et al* [11, 12] reported a preference for NCCN guidelines than ASCO guidelines in a survey among oncologists. Most institutions adopted NCCN and Sub-Saharan NCCN guidelines as the local guidelines with a few modifications. However, our study reported infrequent usage of these local guidelines amongst the centres that had dedicated institution-based guidelines. This may be attributed to lack of awareness among all cadres of staff or non-familiarity with the local guidelines as reported by Cabana *et al* [13] or due to lack of confidence in the local guidelines as reported by Tunis *et al* [9]. The reason for infrequent use of the local guidelines was not reflected in this study, future studies may have to explain this.

Respondents generally used the breast cancer guidelines to access evidence-based treatment protocol and comprehensive/multi-disciplinary management decisions. This study reveals a knowledge gap between the Low- and Medium-income (LMIC) countries and developed countries who focus on ensuring standardised care and improved treatment outcome as reported by Dillmon *et al* [11], Hebert-Croteau *et al* [14] and Lash *et al* [15] in their studies. This suggests that adherence to breast cancer guidelines may improve patient outcome and enhance quality of care.

This study found more guideline usage in government hospitals and teaching hospital settings (81.6%) than in the private setting though only a few of the respondents are involved in private practice. The practitioners in the private setting (4.9%) were all consultants and they also reported using the guidelines. The predominant use seen in government and teaching hospitals may be attributed to the fact that MDT meetings and research are more often practiced in the teaching hospitals which are academic environs. In the private hospital setting, guidelines were used relatively more commonly owing to the fact that most patients attending such facilities can afford recommended treatments as seen in the guidelines to a reasonable extent, when compared to those in the government hospitals. This is similar to what was reported by Polsa *et al* [16].

The major barriers to adherence to guideline use according to this study are non-compatibility with available resources and its complexity of use. Although the latter has been reported by Cabana [13] in an earlier study as a barrier, non-compatibility may be found mostly in LMIC countries where most patients are not on health insurance and the available health insurance scheme has little or no cancer care coverage [17]. A study by Mustapha *et al* [18] on cervical cancer patients in Nigeria showed all of the 78 patients paying out of pocket with none on health insurance and suffering attendant financial burden. Financial toxicity therefore serves as a major barrier for most oncologists as most recommendations in the guidelines are not easily accessible and/or affordable. Studies by Kerr *et al* [12] and Eccles *et al* [19] also highlighted this as a major barrier. Attempts made to combat this include the introduction of the Sub-Saharan version/Institution adopted guidelines, though this has not yielded the desired result as this study has revealed low use of these guidelines. The study however did not inquire for the reason for low use. And this is one limitation of our results. Another significant barrier was the fact that the guidelines may not make provision for individualised treatment needed in some instances when the case is not well captured or specified in the guidelines. The oncologist is thus left with the decision of modifying documented guidelines to suit that moment [20].

## Conclusion

The level of adherence to breast cancer treatment guidelines among oncologists in Nigeria varies in actual practice. The use of the adapted Sub-Saharan version and institution developed guidelines designed to suit this target population is below what is expected and more awareness has to be created for it. Unaffordability still emerged as a major limitation to standardised care and adherence to treatment guidelines in Nigeria. There is a need to ensure health insurance coverage for all, including oncology care, as this will likely play a significant role in achieving best standard practice.

## Conflicts of interest and funding

The authors wish to declare no conflict of interest as the research received no sponsorship or financial support from any individual or organisation.

## Figures and Tables

**Table 1a.** Respondents’ socio-demographic characteristics.

	Frequency (*n* = 103)	Percentage (%)
Age group (years)		
20–29	3	2.9
30–39	42	40.8
40–49	43	41.7
50–59	11	10.7
≥60	4	3.9
Median age: 41.5 years		
Sex		
Male	71	68.9
Female	32	31.1
Marital status		
Single	8	7.8
Married	95	92.2
Level		
Junior resident	12	11.7
Senior resident	27	26.2
Consultant	64	62.1
Speciality		
Radiation/oncology	78	75.7
Surgical oncology	25	24.3
Duration (years)		
<5	17	16.5
5–10	35	34
>10	28	27.2
Resident-in-training	23	22.3
Location of practice		
University hospital	72	69.9
Private hospital	5	4.9
Pharmaceutical organisation	2	1.9
Government hospital	22	21.4
Federal medical centre	2	2
Private practice done		
Yes	49	47.6
No	54	52.4
Had training outside the country in the past		
Yes	54	52.4
No	49	47.6
Training length (month) (*n* = 54)		
<1	3	5.6
1–5	29	53.7
6–12	11	20.4
>12	10	18.5
Not indicated	1	1.9

**Table 1b.** Respondents’ socio-demographic characteristics.

State of practice	Frequency (*n* = 103)	Percentage (%)
Akwa Ibom	1	1
Benue	1	1
Ebonyi	1	1
Edo	4	3.9
Ekiti	1	1
Enugu	6	5.8
FCT	13	12.6
Gombe	1	1
Imo	1	1
Kaduna	4	3.9
Kano	1	1
Katsina	1	1
Kebbi	1	1
Lagos	37	35.9
Ogun	2	1.9
Ondo	4	3.9
Osun	6	5.8
Oyo	10	9.7
Plateau	3	2.9
Rivers	1	1
Sokoto	4	3.9

**Table 2.** Knowledge of breast cancer guidelines.

First learnt about the breast cancer guideline from	Frequency (*n* = 103)	Percentage (%)
Consultant/mentor	39	37.9
Colleague	16	15.5
Conferences/clinical meetings	33	32
Online search	10	9.7
Institution protocol	5	4.9

**Table 3.** Responses to selected questions.

	Frequency (*n* = 103)	Percentage (%)
How often do you use these guidelines? (e.g. in a week)		
Always (more than 10 times)	28	27%
Often (4–10times)	40	39%
Rarely (less than 2 times)	10	10%
Sometimes (2–3 times)	25	24%
Primary reason for referring to a guideline (single response)		
Evidence based results	68	66
Comprehensive report	4	3.9
Multidisciplinary opinion	11	10.7
Applicable to target population	8	7.8
Easy to navigate through	1	1
Accessibility on the web	2	1.9
Tied to reimbursement	0	0
Updates on latest findings	9	8.7
Other reasons for referring to a guideline (multiple responses)		
Evidence based results	47	45.6
Comprehensive report	44	42.7
Multidisciplinary opinion	43	41.7
Applicable to target population	26	25.2
Easy to navigate through	33	32
Accessibility on the web	29	28.2
Tied to reimbursement	1	1
Updates on latest findings	43	41.7

**Table 4.** Respondents’ patients use of guidelines.

	Frequency (*n* = 103)	Percentage (%)
Group of patient guidelines are often used for		
Private patients	19	18.4
General hospital patients	84	81.6
Why use guidelines for private patients (*n* = 19)		
Fewer patients, so more time to consult	7	36.8
Patients can afford the guideline-based treatment	18	94.7
Patients are usually more educated or exposed	6	31.6
To provide top-notch services	9	47.4
Not applicable	0	0
Other	0	0

**Table 5.** Benefits of using breast cancer guidelines.

Benefits	Frequency (*n* = 103)	Percentage (%)
Encourage high quality, evidence-based management	92	89.3
Allows for standardised care	95	92.2
Improves patient outcome	73	70.9
Reduces health disparities	48	46.6
Reduces medico-legal risk	55	53.4
Curb mal-practices or mismanagement	58	56.3
Improve mid-level care givers (General practitioners/ Family physicians/others) practices	34	33
Improves my confidence in managing breast cancer patients	57	55.3
Others	2	0.19
Factors that would increase use of breast cancer guidelines		
Improved accessibility to guidelines	70	68
Fewer patient per clinic	39	37.9
Institution based guidelines/protocol	76	73.8
Affordable cancer care/health insurance coverage	72	69.9
More multidisciplinary meetings	57	55.3
More training/education on use of guidelines	57	55.3

**Table 6.** Barriers to guidelines use.

Barriers to guidelines use	Frequency (*n* = 103)	Percentage (%)
Unaware of any such guidelines	**19**	**18.4**
Inaccessibility of these guidelines	**22**	**21.4**
They are too complex	**25**	**24.3**
The contents are not beneficial/have low applicability to my patients	**15**	**14.6**
Not compatible with available resources	**58**	**56.3**
Not enough time due to patient overload	**23**	**22.3**
Hinders individualised care in some unique circumstances	**27**	**26.2**
Lack of internet access	**14**	**13.6**
They are incomplete	**0**	**0**
They are biased	**2**	**1.9**
They are outdated	**1**	**1**
I don’t agree with the management outlined in the guidelines	**1**	**1**
I trust my personal knowledge and experience	**1**	**1**

**Table 7a.** Association between frequency of breast cancer guideline usage and age group.

Age group	Frequency of breast cancer guideline use				Chi square (*X*^2^)
	Rarely	Sometimes	Often	Always	
20–29	2 (20.0%)	0 (0.0%)	1 (2.5%)	0 (0.0%)	*X^2^* = 29.02
30–39	3 (30.0%)	16 (64.0%)	11 (27.5%)	12 (42.9%)	*df* = 12
40–49	3 (30.0%)	6 (24.0%)	19 (47.5%)	15 (53.6%)	*p* = 0.004
50–59	2 (20.0%)	3 (12.0%)	5 (12.5%)	1 (3.6%)	
≥60	0 (0.0%)	0 (0.0%)	4 (10.0%)	0 (0.0%)	

**Table 7b.** Association between frequency of breast cancer guideline usage and marital status.

Marital status	Frequency of breast cancer guideline use				Chi square (*X^2^*)
	Rarely	Sometimes	Often	Always	
Single	3 (30.0%)	2 (8.0%)	1 (2.5%)	2 (7.1%)	*X^2^* = 8.47
Married	7 (70.0%)	23 (92.0%)	39 (97.5%)	26 (92.9%)	*df* = 3
					*p* = 0.037

**Table 7c.** Association between use of a breast cancer guideline to make a treatment decision and level.

Level	Have used a breast cancer guideline to make a treatment decision		Chi square (*X^2^*)
	Yes	No	
Junior resident	8 (8.4%)	4 (50.0%)	*X^2^* = 14.81
Senior resident	24 (25.3%)	3 (37.5%)	*df* = 2
Consultant	63 (66.3%)	1 (12.5%)	*p* = 0.001

**Table 7d.** Association between frequency of breast cancer guideline use and speciality.

Speciality	Frequency of breast cancer guideline use				Chi square (*X^2^*)
	Rarely	Sometimes	Often	Always	
Radiation/oncology	2 (20.0%)	18 (72.0%)	34 (85.0%)	24 (85.7%)	*X^2^* = 20.48
Surgical oncology	8 (80.0%)	7 (28.0%)	6 (15.0%)	4 (14.3%)	*df* = 3
					*p* = 0.000

**Table 7e.** Association between use of breast cancer guideline to make a treatment decision and having a training abroad.

Had a training abroad	Have used a breast cancer guideline to make a t reatment decision		Chi square (*X^2^*)
	Yes	No	
Yes	53 (55.8%)	1 (12.5%)	*X^2^* = 5.54
No	42 (44.2%)	7 (87.5%)	*df* = 1
			*p* = 0.019
